# No difference in load to failure or stiffness between transosseous tunnels, suture anchors, and cortical buttons for pectoralis major tendon repair: a systematic review & meta-analysis

**DOI:** 10.1186/s40634-023-00617-9

**Published:** 2023-05-26

**Authors:** Sean B. Sequeira, Lynette M. Sequeira, Mark D. Wieland, Casey M. Imbergamo, Kenneth Tepper

**Affiliations:** Department of Orthopaedic Surgery, MedStar Union Memorial Hospital, 3333 North Calvert, Street, Suite 400, Baltimore, MD 21218 USA

**Keywords:** Pectoralis major tendon repair, Bone trough, Suture anchor, Biomechanics, Cadaveric studies

## Abstract

**Purpose:**

Surgical options for pectoralis major tendon tears include primary repair, though there is no consensus as to which constructs are biomechanically superior for repair.

**Methods:**

A systematic review was performed by searching PubMed, the Cochrane library, and Embase using PRISMA guidelines to identify studies that analyzed the biomechanical properties of bone tunnels (BT), cortical buttons (CB) and suture anchors (SA) techniques for pectoralis major tendon repair. The search phrase implemented was ‘pectoralis major tendon repair biomechanics’. Studies that did not evaluate biomechanical outcome data, evaluated partial pectoralis major tendon tears, and non-English articles were excluded. Evaluated outcomes included ultimate load to failure (N) and stiffness (N/mm).

**Results:**

Six studies met inclusion criteria, including a total of 124 cadaveric specimens, for pectoralis major tendon repair comparing BT with SA and CB. Pooled analysis from four studies reporting on ultimate load to failure between BT and SA failed to reveal a difference between BT and SA (*p* = 0.489). Pooled analysis from two studies reporting on stiffness failed to reveal a difference in favor of BT compared to SA (*p* = 0.705). Pooled analysis from four studies reporting on ultimate load to failure between BT and CB failed to reveal a difference between BT and CB (*p* = 0.567). Pooled analysis from two studies reporting on stiffness failed to reveal a difference in favor of BT compared to CB (*p* = 0.701).

**Conclusions:**

There was no difference in load to failure or stiffness when using BT, CB, or SA in pectoralis major tendon repairs. This review reveals that clinical outcomes may better inform which fixation construct to implement in pectoralis major tendon repairs.

**Level of evidence:**

I.

## Introduction

The pectoralis major tendon is a powerful adductor and internal rotator of the arm and is generally divided into a clavicular and sternocostal segment. Tendon ruptures are rare occurrences, but most commonly occur during lifting activities, the bench press exercise, that require contraction with the arm in extension and external rotation [10]. Since the tendon confers substantial function to activities of daily living, most surgeons opt for operative treatment of tendon ruptures unless the patient is a poor candidate for surgery [3]. Operative treatment classically involves primary repair, though there are several fixation constructs available to reattach the tendon to its anatomic humeral insertion.

The historical gold standard for tendon fixation in pectoralis major tendon ruptures is the creation of transosseous bone tunnels, sometimes aided by bone troughs [12]. Tranosseous bone tunnels have demonstrated strong clinical results and high postoperative functional scores [3]. Nevertheless, the creation of tranosseous bone tunnels, especially with bone troughs, have come under scrutiny due to the creation of a stress riser during trough creation, in order to dock and secure the tendon [14].

Suture anchors are a relatively new fixation construct that have been utilized for tendinous and ligamentous fixation in many other anatomic regions of the musculoskeletal system. Aarimaa et al. performed a retrospective study and found no difference in clinical outcomes between Depuy Mitek G2 anchors and the use of tranosseous bone tunnels [1]. Suture anchors offer the theoretical biomechanical benefit of eliminating the need to drill a socket for tendon docking and can directly fix the tendon to its humeral native insertion. Despite these benefits, suture anchors have been called into question due to their cost and the integrity of the fixation is solely dependent on the anchor [15]. Most recently, cortical buttons (CB) have been introduced as yet another fixation construct to reattach avulsed tendon to its native insertion on the proximal humerus [12].

Several studies have examined the biomechanical effects of transosseous bone tunnels, suture anchors, and cortical buttons for pectoralis major tendon repair, though the biomechanical superiority of bone tunnels versus suture anchors versus cortical buttons has yet to be identified in a comprehensive review – in isolation, these studies either do not compare all three fixation devices, are poorly powered, and/or do not include major biomechanical outcomes. The purpose of this study was to systematically review the existing literature to evaluate the biomechanical properties of bone tunnels (BT) versus suture anchor (SA) versus cortical buttons (CB) techniques for pectoralis major tendon repair. The null hypothesis was that there would be no difference in biomechanical parameters between BT, SA, and CB for pectoralis major tendon repair.

## Methods

Preferred Reporting Items for Systematic Reviews and Meta-Analyses (PRISMA) guidelines were utilized to evaluate studies within the literature for inclusion within this systematic review and meta-analysis. A cohort of two independent reviewers searched the PubMed, Embase, and Cochrane Library databases from January 1^st^, 2000 to June, 30^th^ 2022. The electronic search string utilized was ‘pectoralis major tendon repair.’ Human, animal, and sawbone cadaveric studies that assessed the biomechanics of pectoralis major tendon repair with tranosseous bone tunnels and/or suture anchor were included within the review. Exclusion criteria were limited to cadaveric studies performed in vivo, studies evaluating partial tendon tears, studies that evaluated repairs or reconstructions of soft tissue structures other than the pectoralis major, clinical studies, and studies without full text available. Studies in vivo were studies in which the soft tissue surrounding the pectoralis major were preserved and were performed in intact cadavers. Data extraction from each study was performed independently and reconciled by a third author. There was no need for funding or a third party to obtain any collected data.

In order to evaluate bias and legitimacy of each cadaveric study, The Quality Appraisal for Cadaveric Studies (QUACS) scale was utilized [17]. The scale consists of a checklist encompassing 13 items. Each is to be scored with either 0 (no/not stated) or 1 (yes/present) point which evaluates methodological bias. Points are only assigned if a criterion is met without any doubt, and a final percentage is given as the total score. Scores above 75% were deemed acceptable and were included within the study.

All outcomes evaluated were biomechanical in nature and included: ultimate load to failure (N) and stiffness (N/mm). Of the six studies that evaluated pectoralis major tendon repair, four evaluated ultimate load to failure between BT and SA and four commented on ultimate load to failure between BT and CB. Two studies commented on stiffness comparing BT and SA and two other studies commented on stiffness between BT and CB.

In the event that standard deviation measurements were excluded from a study’s analysis, the methodology described in the Cochrane Handbook for Systematic Reviews of Interventions (version 6.2.0) was utilized. Once all means and standard deviations were collected/computed, weighted averages were calculated for all quantitative outcomes. The outcomes were summarized in a forest plot when data from 2 or more studies were available. Using a random-effects model, standardized mean differences (SMD) with 95% confidence intervals (CI) were calculated and embedded within the forest plot. A random-effects model was used in order to incorporate the heterogeneity between each included study into the final statistical analysis. In order to quantify the degree of heterogeneity due to between-study characteristics, *I*^*2*^ statistics were computed. Meta-analyses statistics and generation of forest plots figures were performed using OpenMetaAnalyst, which implements metafor R console code.

## Results

**Fig. 1:**
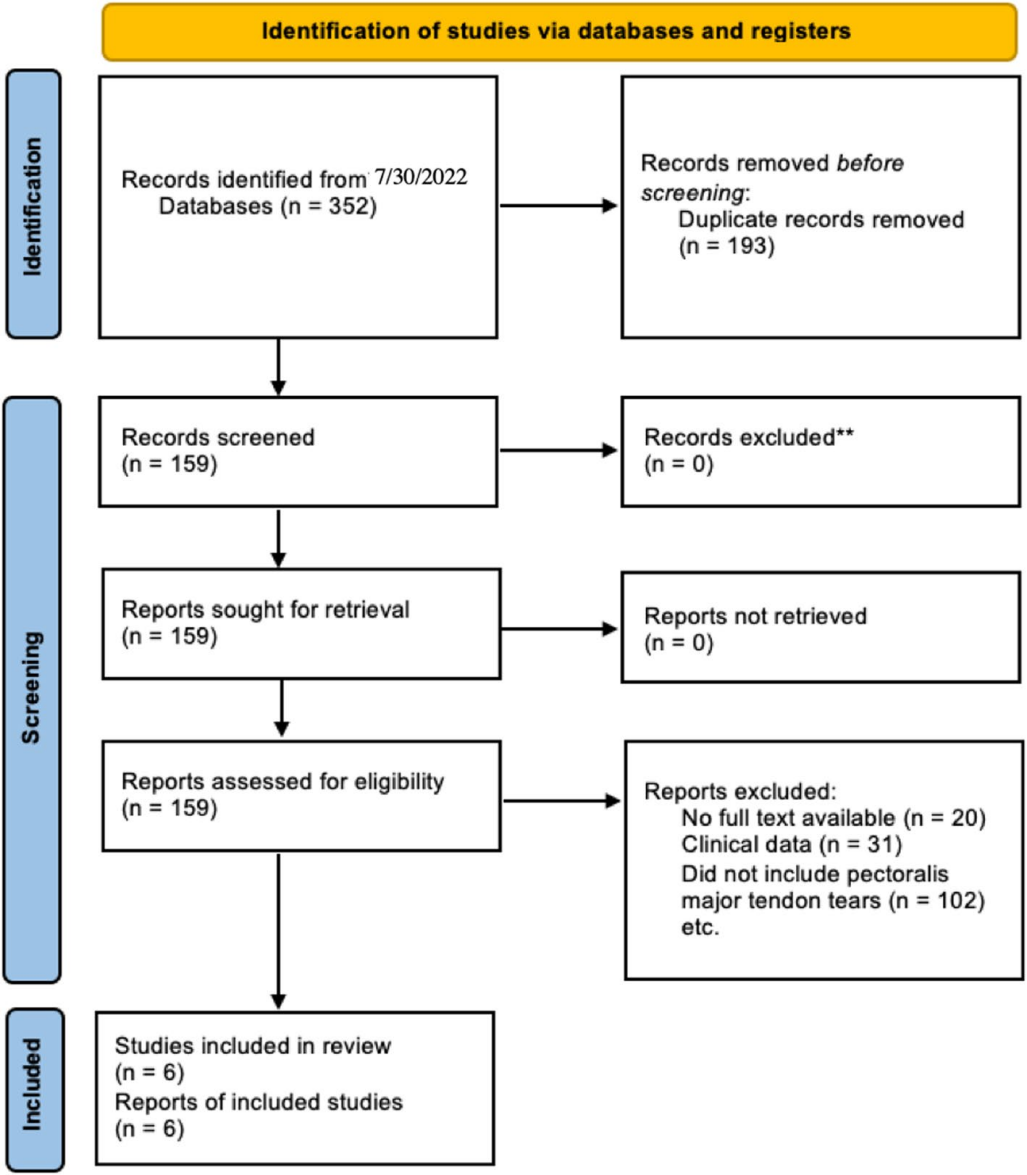
PRISMA flow chart detailing the database search and inclusion/exclusion process

A total of 352 studies were reviewed by title and/or abstract to determine study eligibility based on aforementioned inclusion criteria (Fig. 1). Six studies, including a total of 124 cadaveric specimens, met inclusion criteria for pectoralis major tendon repair. These studies are summarized in Table 1.

**Table 1:** Fixation details of included studies

Study	Techniques compared & sample size	Cadaveric age	Anchors used	Number of tunnels	Number of anchors	Sutures used in tunnel	Number of buttons used	Type of button	Ultimate load to failure (N)	Stiffness (N/mm)	Methodologic quality assessment of included studies
Hart et al	6 SA; 6 BT	85 ± 3.49 years	Mitek GII Superanchors	4	4	2 #2 Orthocord sutures/tunnel; 1 Krackow and 1 Bunnell stitches/tunnel	N/A	N/A	+	+	**MCMS**
Rabuck et al	10 SA; 10 BT; 10 endobutton	54.4 (27–68) years	5 mm, PEEK Biocorkscrew, Arthrex Inc	4	3	3 No.2 Fiberwire, Arthrex	3	Titanium Pec Button, Arthrex	+	-	84.6%
Sherman et al	8 SA; 8 BT; 8 endobutton	73.1 years	5.5 mm Super QuickAnchors, Depuy Mitek	4	3	No. 2 Orthocord (Depuy Mitek)	3	Titanium Pec Button, Arthrex	+	+	92.3%
Edgar et al	8 BT; 8 endobutton	64.61 ± 7.62 years	N/A	4	N/A	2 No. 2 Fibrewire suture/tunnel	3	Pec Button, Arthrex	+	+	84.6%
Wilson et al	10 BT; 10 screw; 10 SA	NR	2.8 mm Lupine Loop Anchor, Depuy Mitek	4	3	N/A	N/A	N/A	+	-	100%
Thomas et al	11 BT; 11 endobutton	8 months	N/A	4	N/A	2 No. 2 Fibrewire suture/tunnel	1 with 2 No. 2 Fibrewire suture	Pec Button, Arthrex	+	-	76.9%

### Specimen preparation

In all studies, the pectoralis major tendon was visualized through superficial and deep dissection and sharply avulsed from its tendinous insertion on the proximal humerus. Both the clavicular and sternocostal heads were sharply lifted from their respective insertions on the proximal humerus.

### Transosseous BT surgical technique

All six studies included a BT surgical technique. Five out of six studies created a bone trough to aid in the tranosseous tunnel formation; Sherman et al. did not utilize a bone trough in their transosseous tunnel surgical technique. Three of those five studies created a bone trough of 4 cm, one study created a bone trough of 5 cm, and one study [16] created a 10 × 20 × 2 mm trough. Four studies utilized a 2 mm drill bit to create tunnels, while one study [16] used a 3.2 mm drill. Four studies created four tunnels one cm lateral to the created trough [6, 11, 16, 18]. Sherman et al. drilled two tunnels 5 mm proximal and distal to the insertion and two tunnels were placed between the aforementioned two tunnels [13]. Edgar et al. created three tunnels and passed suture ends, that were previously whipstitched to the tendon, through the tunnels lateral to the native tendon footprint [4]. In five of the six studies that utilized a bone trough, the tendon was inserted into the trough, shortening the working length of the repair construct. Sutures were whipstitched to the tendon prior to insertion of the tendon through the transosseous tunnel in four studies. Sherman et al. sutured to tendon using the Mason-Allen configuration [13].

### SA surgical technique

Four studies included the SA surgical technique [6, 11, 13, 18]. In those studies, the native tendinous insertion on the proximal humerus was lightly abraded. Two mm drills were used to place anchors over a four cm area. Suture anchors were loaded with suture and one limb was passed through released pectoralis tendon. Three studies used Krackow stitches to pass one limb of the suture through the tendon, while one study [13] secured the suture using a modified Mason-Allen stitch configuration. The second arm, in all studies, was used to tension the tendon before securing the repair.

### CB surgical technique

Four studies included the CB surgical technique [4, 11, 13, 16]. In those studies, a 3.2 mm drill was used to create 3 unicortical holes for cortical button placement. In one study, a 10 × 20 × 2 mm trough was created prior to drilling to facilitate the passage of the cortical button [16]. Sutures were secured to the tendon either by Krackow locking stitches [4, 11, 16] or Mason Allen configuration [13].

#### Methodological quality – QUACS score

The risk of bias and methodological quality of the included studies were assessed using the QUACS scale, which has been previously validated. The mean QUACS score was 86.25% (range 76.9%-100%). All six studies satisfied the threshold for a satisfactory methodological quality (> 75%).

### Ultimate load to failure

**Fig. 2:**
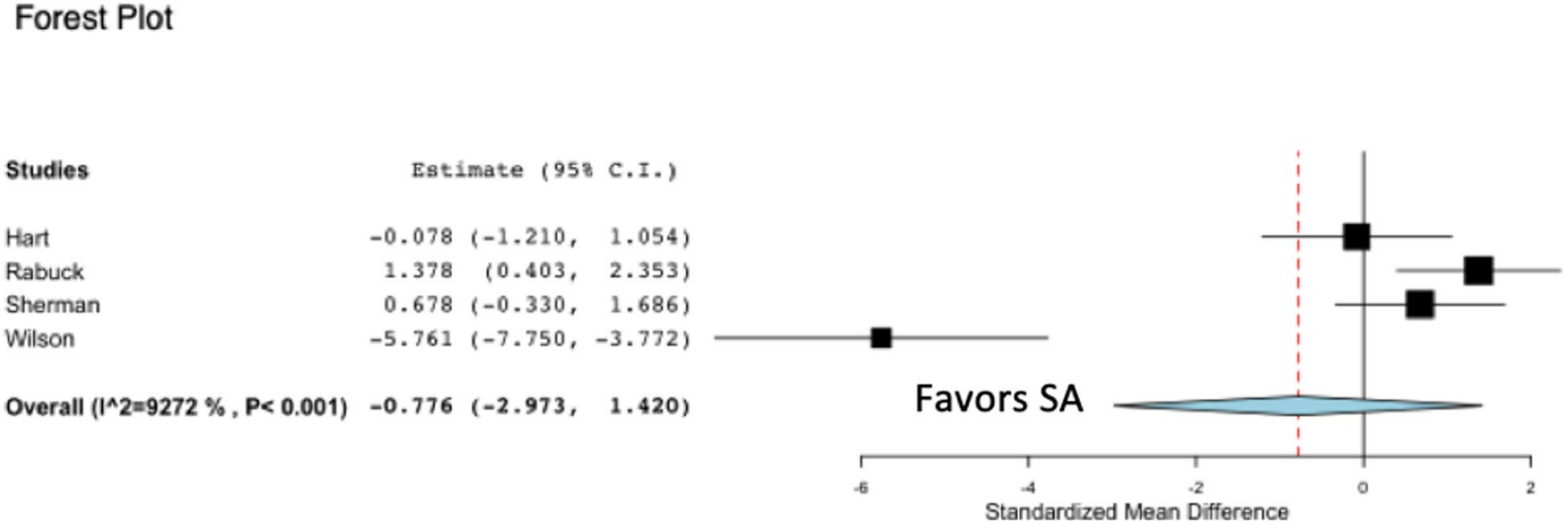
Forest plot demonstrating a standardized mean difference of ultimate load to failure (N) in favor of BT pectoralis major tendon repair compared to SA

Four of six studies on pectoralis major tendon repair reported on ultimate load to failure (N), comparing BT and SA [6, 11, 13, 18] (Fig. 2). Two out of the four studies concluded that the use of BT in tendon repair exhibited higher loads to failure, while the remaining studies concluded that there was no difference in ultimate load to failure between BT and SA. The pooled analysis from four studies reporting on ultimate load to failure for pectoralis major tendon repair failed to reveal a statistically significant difference in favor of BT compared to SA (*p* = 0.489). Heterogeneity, as calculated by the I^2^ statistic, was determined to be 92.7% for the pooled studies evaluating ultimate load to failure between BT and SA.

**Fig. 3:**
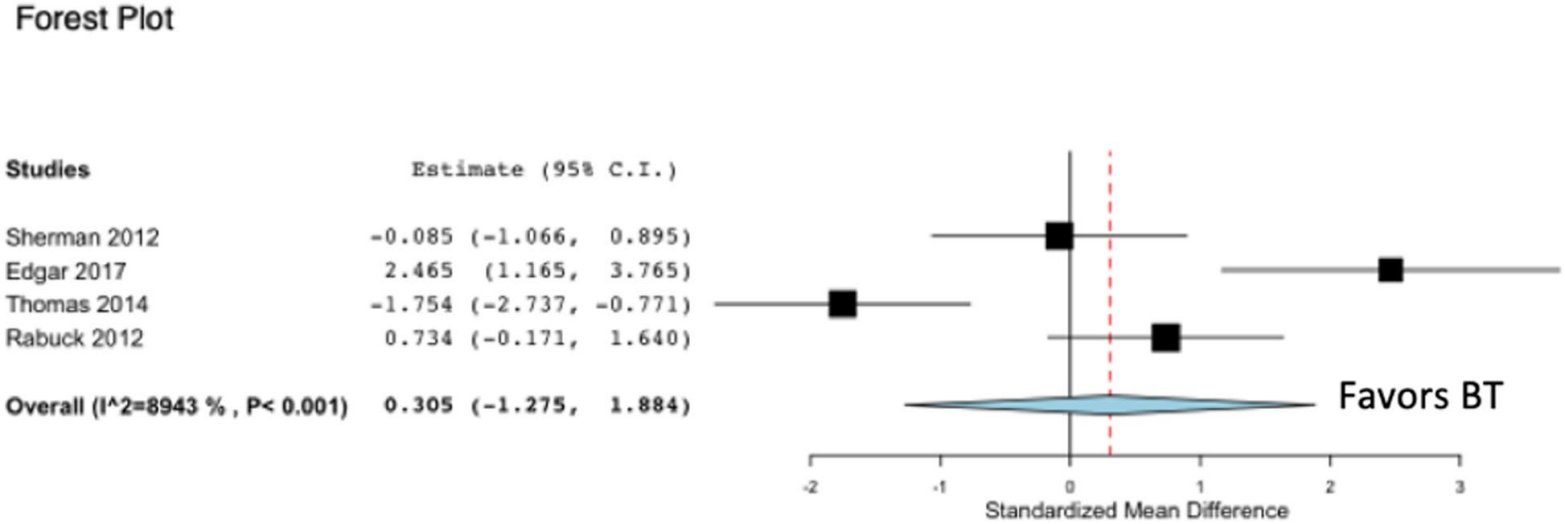
Forest plot demonstrating a standardized mean difference of ultimate load to failure (N) in favor of BT pectoralis major tendon repair compared to CB

Four of six studies on pectoralis major tendon repair reported on ultimate load to failure (N), comparing BT and CB [4, 11, 13, 16] (Fig. 3). One of the four studies concluded that the use of CB in tendon repair exhibited higher loads to failure [4] and one study concluded that the use of CB exhibited lower loads to failure compared to BT [16], while the remaining studies concluded that there was no difference in ultimate load to failure between BT and CB. The pooled analysis from four studies reporting on ultimate load to failure for tendon repair failed to reveal a statistically significant difference in favor of BT compared to CB (*p* = 0.705). Heterogeneity, as calculated by the I^2^ statistic, was determined to be 89.4% for the pooled studies evaluating ultimate load to failure between BT and CB.

### Stiffness

**Fig. 4:**
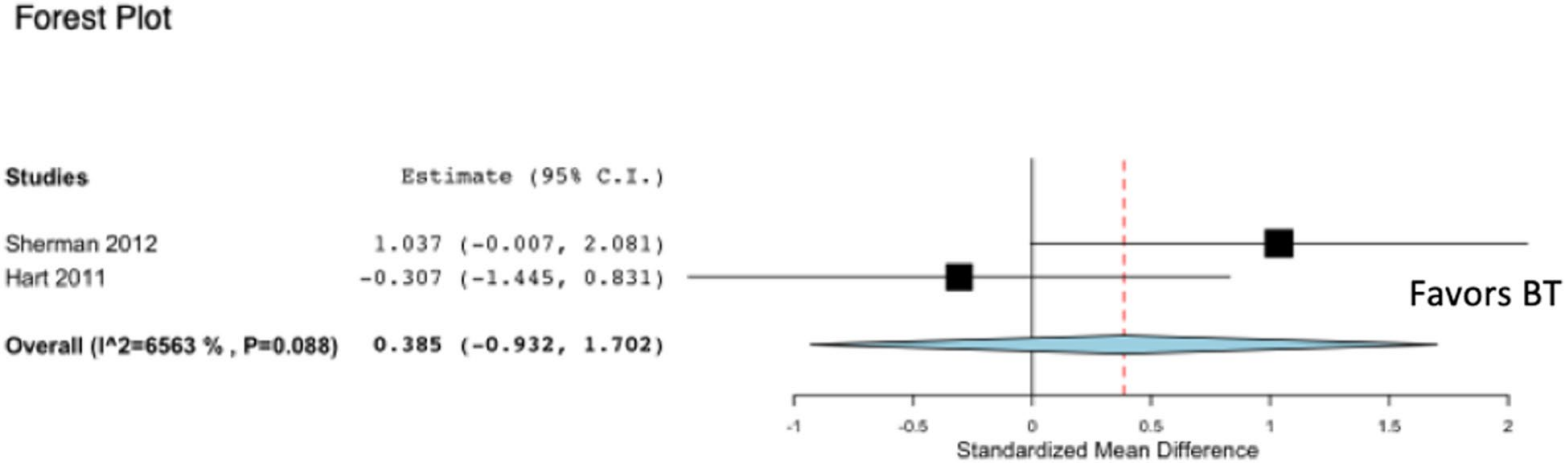
Forest plot demonstrating a standardized mean difference of ultimate load to failure between BT and SA following pectoralis major tendon repair

Two out of six studies on pectoralis major tendon repair reported on stiffness (N/mm) between BT and SA [6, 13] (Fig. 4). Neither of these studies that evaluated stiffness of the two fixation constructs, BT and SA, found a difference between BT and SA following pectoralis major tendon repair. The pooled analysis from two studies reporting on stiffness for pectoralis major tendon repair failed to reveal a statistically significant difference in favor of BT compared to SA (*p* = 0.567). Heterogeneity, as calculated by the I^2^ statistic, was determined to be 65.6% for the pooled studies evaluating stiffness between BT and SA.

**Fig. 5:**
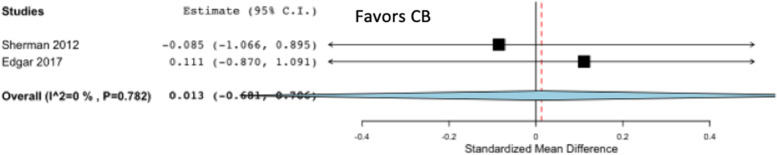
Forest plot demonstrating a standardized mean difference of ultimate load to failure between BT and CB following pectoralis major tendon repair

Two out of six studies on pectoralis major tendon repair reported on stiffness (N/mm) between BT and CB [4, 13] (Fig. 5). Neither of these studies that evaluated stiffness of the two fixation constructs, BT and CB, found a difference between BT and CB following pectoralis major tendon repair. The pooled analysis from two studies reporting on stiffness for pectoralis major tendon repair failed to reveal a statistically significant difference in favor of BT compared to CB (*p* = 0.971). Heterogeneity, as calculated by the I^2^ statistic, was determined to be 0% for the pooled studies evaluating stiffness between BT and CB.

## Discussion

After evaluating the current literature for biomechanical properties of transosseous bone tunnels and suture anchors for primary repair of pectoralis major tendon ruptures, our findings suggest that there is no difference in ultimate load to failure and stiffness between BT and SA in pectoralis major tendon repair. These findings may suggest that the choice between various fixation constructs should rely on clinical data and functional outcome scores, especially since biomechanical comparisons between BT and SA are equivocal.

Overall, clinical and functional outcome studies on various fixation constructs used to repair pectoralis major tendon ruptures demonstrate that there is little difference between BT, CB and SA. For example, in a study by Antosh et al., the use of BT for tendon repair was associated with good to excellent overall DASH, Work Module, and Sports Module scores [2]. In a study by Garrigues et al., they found that the use of transosseous tunnels was associated with restoration of average preinjury bench press, improvement in Penn Shoulder Score, American Shoulder & Elbow Surgeons (ASES) score, and Single Assessment Numeric Evaluation scores (SANE) [5]. In a study that evaluated the use of SA for tendon fixation, Kakwani et al. concluded that SA was associated with return to sports in 8.5 months and 11/12 patients with excellent or good functional outcomes [7]. Finally, a retrospective review of twenty patients that underwent SA fixation by a single surgeon found that the use of SA during pectoralis major tendon repair is associated with high patient satisfaction and predictable return of strength and function, especially when compared to alternative fixation methods [9]. Furthermore, cortical buttons similarly achieve excellent clinical and functional outcomes – in a study by Kang et al., twelve recreational athletes were treated with button fixation and achieved high ASES and SANE scores, not to mention a return to isokinetic strength in the affected shoulder [8].

In a large meta-analysis of clinical studies evaluating patient reported outcome measures and outcomes using different fixation constructs, there were no significant differences in functional outcome score, range of motion, full isometric strength, return to activity, between BT, CB and SA [3]. Furthermore, there was no significant difference in Single Assessment Numeric Evaluation Score (SANE), ASES score, DASH score, complication rate, or overall satisfaction rate. This study, though clinical, is consistent with our findings in that both clinically and biomechanically, there is no difference between BT, CB and SA for pectoralis major tendon repair. Notably, this study only compiled clinical outcomes and data in a large systematic review, thereby making our biomechanical systematic review and meta-analysis novel.

Though this study has many strengths that make it a worthwhile addition to the literature, it also has its limitations. Only six studies were included in this review, which may suggest that some of the conclusions drawn herein are not adequately powered. Furthermore, the techniques by which ultimate load to failure and stiffness were evaluated exhibited a degree of heterogeneity between studies and, therefore, the results and our concomitant conclusions should be interpreted with caution. Similarly, the quantitative heterogeneity, as represented by *I*^*2*^ statistic, revealed that both ultimate load to failure and stiffness for tendinous fixation demonstrated considerable heterogeneity, all of which should be considered when interpreting our findings.

Nevertheless, our review evaluated the existing literature to comprehensively analyze the biomechanical properties of three commonly tested techniques for pectoralis major tendon repair, a topic not previously studied in the literature. Though our findings should be interpreted with caution, they nevertheless provide important insight into the biomechanical properties of these fixation constructs and help to better define the optimal techniques and constructs that are available in the treatment of tendon rupture. Though there is no appreciable biomechanical difference between BT, CB and SA nor do clinical studies reveal a significant difference between the fixation constructs, surgeons should consider and interpret all of these biomechanical and clinical findings to best inform their chosen repair technique and construct for tendon repair intraoperatively.

## Conclusion

Our review of the biomechanics literature that evaluated ultimate load to failure and stiffness between BT, SA, and CB for pectoralis major tendon repair demonstrates that there is no difference between the three constructs for fixation of pectoralis major tendon repairs. Surgeons should rely on clinical and functional results of various fixation strategies to determine which construct confers better outcomes for tendon repair.

## Data Availability

The datasets used and/or analyzed during the current study are available from the corresponding author on reasonable request. Furthermore, all data used in this study was publicly available within each study included within this meta-analysis.
